# Application of artificial neural network for the quality-based classification of spray-dried rhubarb juice powders

**DOI:** 10.1007/s13197-020-04537-9

**Published:** 2020-05-30

**Authors:** K. Przybył, J. Gawałek, K. Koszela

**Affiliations:** 1 Institute of Biosystems Engineering, Poznan University of Life Sciences, Wojska Polskiego 50, 60-625 Poznan, Poland; 2 Food Engineering Group, Institute of Food Technology of Plant Origin, Food Sciences and Nutrition, Poznan University of Life Sciences, Wojska Polskiego 31/33, 60-624 Poznan, Poland

**Keywords:** Vegetable powders, Image analysis, Spray-drying, Classification, Artificial neural network (ANN)

## Abstract

The aim of the study was to develop a neural model enabling classification of fruit spray dried powders, on the basis of graphic data acquired from a bitmap received in the process of spray drying. The neural model was developed with multi-layer perceptron topology. Input variables were expressed in 46 image descriptors based on RGB, YCbCr, HSV (B) and HSL models. Sensitivity analysis of input variables and principal component analysis determined the significance level of each attribute. The optimal model with the lowest error value root mean square, at the level of 0.04 contained 46 neurons in the input layer, 11 neurons in the hidden layer, 10 neurons in the output layer. The results allowed to show that dyeing force (color features) had influence on effective differentiation of the research material consisting of spray-dried powders of rhubarb juice with various dried juice content levels: 30, 40 and 50% as well as high (“H”) and low (“L”) level of saccharification a chosen carrier (potato maltodextrin).

## Introduction

Fruits and vegetables play a major role in human nutrition, mainly due to the fact that they are rich in vitamins, minerals and precious dietary fiber with beneficial effects. According to the reports, fiber decreases cholesterol absorption protecting against cardiovascular diseases. It also lowers the concentration of glucose in the blood, which is especially important for people who suffer from diabetes. Vitamins as well as minerals have a positive effect on organism, for example in cancer and anti-sclerotic prevention, they also have antibacterial properties (Gawałek et al. 2017; Przybył et al. 2018). It happens like that mainly because fruits and vegetables are characterized by low caloric value, almost zero fat presence and high water content. However, water content in vegetables and fruits may lead to their deterioration (Feguš et al. 2015). High level of water activity in fruit and vegetable shortens their shelf life (Fegus et al. 2015). That is why, preservation methods such as, among other things, drying and spray drying are used to reduce the level of water activity. Using proper drying technique, it is possible to eliminate microbiological spoilage of fruits and vegetables and the development of bacterium and other micro-organisms.

In recent times, convenience food is becoming more and more popular. Consumers expect to be able to prepare a meal in a relatively short time, which is also easy to prepare. That kind of food should be characterized, among other things, by high nutritional values, freshness, adequate color, taste, smell and attractive packaging (Augustyńska-Preisner and Ormian 2012). Due to the above and the fact that they are natural components of food, acting as smell, taste and color carrier, instant dishes with fruit and vegetable powders are becoming more and more popular with people. Thus, spray drying seems to be one of the popular ways of producing food powders (Chegini et al. 2008; Gong et al. 2008; Wesołowski and Gawałek 2008), a process characterized by continuity that requires reproducibility of quality of ready-made product.

Quality assessment of the produced powders, carried out in a laboratory, is usually time-consuming and expensive. The above makes it an ineffective ineffective tool of evaluating quality condition of powders during drying process. Fast and precise comparative analysis of forms and colors of powders would enable a swift adjustment of process parameters in case of certain anomalies (deviations from the standard). Carrying out only a full (laboratory) quality assessment of the entire batch of product following manufacturing process means that some products may be rejected (they simply don to meet the requirements and norms), which can lead to financial losses for producer. In order to minimize production costs, producers are looking for modern methods of quality assessment of fruit and vegetable powders obtained through spray drying, which—in the broadly understood aspect of decision-making—are be non-invasive and fast. This study is an initial attempt to analyze spray dried powders obtained from rhubarb juice and their classification based on sensory differences, which in the future may enable to develop such a technique.

Rhubarb (*Rheum rhaponticum L.*) is a plant of the Polygonaceae group (Goula and Adamopoulos 2010). Rhubarb stalk is distinguished by shades of green, red or yellow. It is characterized by ingredients that exhibit high pharmacological and detoxication activity. It also has anti-tumor, diuretic, anti-inflammatory and analgesic effects (Nadulski et al. 2015). The aforementioned health properties of rhubarb are mainly connected with high content of anthocyanins up to 2000 mg kg^−1^ (Zheng et al. 2013). Anthocyanins belong to polyphenolic organic compounds. They are a natural dye of plant origin and have a lot of health-promoting properties, among other things: they protect tissue against excessive UV radiation (Gawałek et al. 2017). Nowadays they are more and more often used in pharmaceutical and cosmetic industry (Takeoka et al. 2013). The presence of anthocyanin compounds in rhubarb is used for effective treatment of cancer cells by its antioxidant effect. (Takeoka et al. 2013).

Application of artificial neural networks (ANN) in issues related to classification and regression issues resulted in the discovery of quality changes concerning food products (Przybył et al. 2018). The method is based on developing neural models, which is relatively faster and more efficient than traditional computing methods. Neural modeling allows to simulate complex processes, e.g. through learning and information processing in a parallel way. One of the major advantages of neural classifiers is the ability to search for pattern classes using the ‘supervised’ learning technique (Gómez-Carracedo et al. 2010). In practice, this constitutes a set of objects, assigned to one class, with the assumption that representative characteristics of individual classes are known prior to implementation of learning process of a model.

The increasing trend of consumer awareness in terms of good nutrition, way of preparation and product quality resulted in the search for new methods of improving quality of product and ways of automating technological processes. Computer image analysis technique supported by artificial intelligence is commonly used as a tool to solve decision-making issues in the field of agricultural engineering (Kumar and Mittal 2008). They are used in research concerning determination of representative characteristics of compost and other agricultural waste, meat and fruit and vegetables: barley, dried carrots, potatoes defects, tomatoes defects (Kozłowski et al. 2016), apple and strawberry sorting, defect sorting in detecting defects in citrus and pedicel/peduncle, and assessment of size of grapevine berries, a system for apple color assessment (Xiaobo et al. 2007), plant leaf detection and recognition of banana fruit maturity (Surya Prabha and Satheesh Kumar 2015), as well as assessment of structural changes in strawberry powders (Przybył et al. 2018). The authors of this paper never came across any cases of use of neural image analysis to identify fruit and vegetable powders in the process of spray drying. However, according to the authors’ knowledge, parameters for assessing quality of powders’ colors are up until now based on methods of colorimetry (Lab) (Modzelewska-Kapituła 2012).

The aim of the study is to develop an multi-layer perceptron (MLP) artificial neural network that classifies selected varieties of rhubarb powders on the basis of information encoded in graphical form—as digital photographs.

## Materials and methods

### Research material

There are many different types of food powders used as flavor, coloring or nutritional components in food processing. The study mainly focuses on comparative analysis and qualification of powders obtained in industrial-scale of spray drying process with different content of dry fruit and vegetable mass in powder and other type of the carrier. Spray drying of fruit and vegetables juices requires the use of the so-called carriers, which are necessary to carry out drying process (Wesołowski and Gawałek 2008). Powders produced in this way contain various concentrations of dry mass of fruit or vegetable juice (carrier constitutes the part of powder) (Gawałek et al. 2017). The research material consists of spray-dried rhubarb juice powders, with various levels of dry juice content: 30, 40 and 50% (potato maltodextrin, used as a carrier, constituted the remaining part of dry powder substance) as well as high (“H”) and low (“L”) level of saccharification carrier. Maltodextrin used during the research has dextrose equivalent (DE) respectively: “H”—DE 26 and “L”—DE 12. Basic physiochemical parameters of powders, which are subject to this research, are within the following range: moisture 1.95–2.80%, water activity 0.14–0.21, bulk density 551–606 kg/m^3^, flowability expressed by Hausner ratio (HR) 1.32–1.42, mean diameter of particles (median D_50_) 16.4–19.1 mm.

The process of image acquisition entailed the use of measurement and research station. The measurement and research station consists of:

- a visible light source in the form of 3 × 70 W (600 lm) lamps and 3 × 60 W (806 lm) and light color 6500 K,
- light tent (0.5 m × 0.5 m × 0.5 m),
- NIKON D5100 SLR camera—image sensor in DX standard,
- lens: Nikkor lens 18-105 mm f/3.5-5.6G VR AF-S DX,
- support stand ARKAS TS01.

According to the standard PN-EN 12464-1: 2004 concerning the uniformness of illumination, the measurement of visible light intensity was taken with CFM DT-1308 luxmeter, on the basis of the calculation of luminance distribution it is concluded that the shadowless chamber complies with requirements concerning uniformness of visible light (VIS).

The images of research material samples are obtained using Nikon D5100 camera with a 16.2-megapixel matrix (CMOS matrix 23.1 × 15.4 mm—DX format), a shadowless tent illuminated by visible light (VIS) of warm white color and color temperature of 6500 Kelvins (K). Color temperature determines color of emitted lights by light source. Illumination is selected on the basis of color of empirical object and is determined quantitatively on the basis of color scale of temperature measured in Kelvins (K). The lower the value of the Kelvin scale, the more can be seen in digital image in orange color. However, light from the end of the scale with higher values is characterized by bluer hue. Parameters of the research station are calibrated in the following way: sensitivity of the camera matrix is set to ISO-125, the aperture is set to f/8, and exposure time is set to 1/125 s; length of the prime lens is set to 70 mm, without flash, white balance is set to manual mode. Following proper adjustment of parameters for each image object and after preparing equipment for image analysis, a database in the form of digital images of the selected types of fruit powders is obtained As a result of the acquisition of empirical data, 127 original 24-bit images in 4928x3264 resolution are obtained.

### Research methodology

The software called Przybyl Image Detector System (‘PIDsystem’), supporting the process of isolating classification features of fruit and vegetable products, was used in the research (Ebrahimi et al. 2012; Przybył et al. 2018).

This software enabled to segment a single image trial and to process many images (Zhao et al. 2016). Next, the images were processed with ‘PIDsystem’ software with image probe module. Using image probe module, the researcher divided a given image into parts in order to divide the bitmap into equal pieces, obtaining approx. 4218 images in 400 × 400 resolution, which were used to construct the training set. An alternative solution, aimed at saving up work time, was the application of module enabling processing of batch image Characteristic features in the form of numerical data from bitmap were obtained with a batch image processing module and saved to a file in csv format. 46 image descriptors were determined for each training set. In order to obtain some of them, the following image conversions had to be carried out:

- image conversion from an RGB (Red Green Blue) model (García-Mateos et al. 2015) to an YCbCr (Y is luma component and CB n CR are the blue-difference and red-difference chroma components) model,
- image conversion from an RGB model to an HSL (Hue Saturation Lightness) model (Philipp and Rath 2002),
- image conversion from an RGB model to an HSV model (Hue Saturation Value) (García-Mateos et al. 2015).

### The following tasks of research

**Fig. 1:**
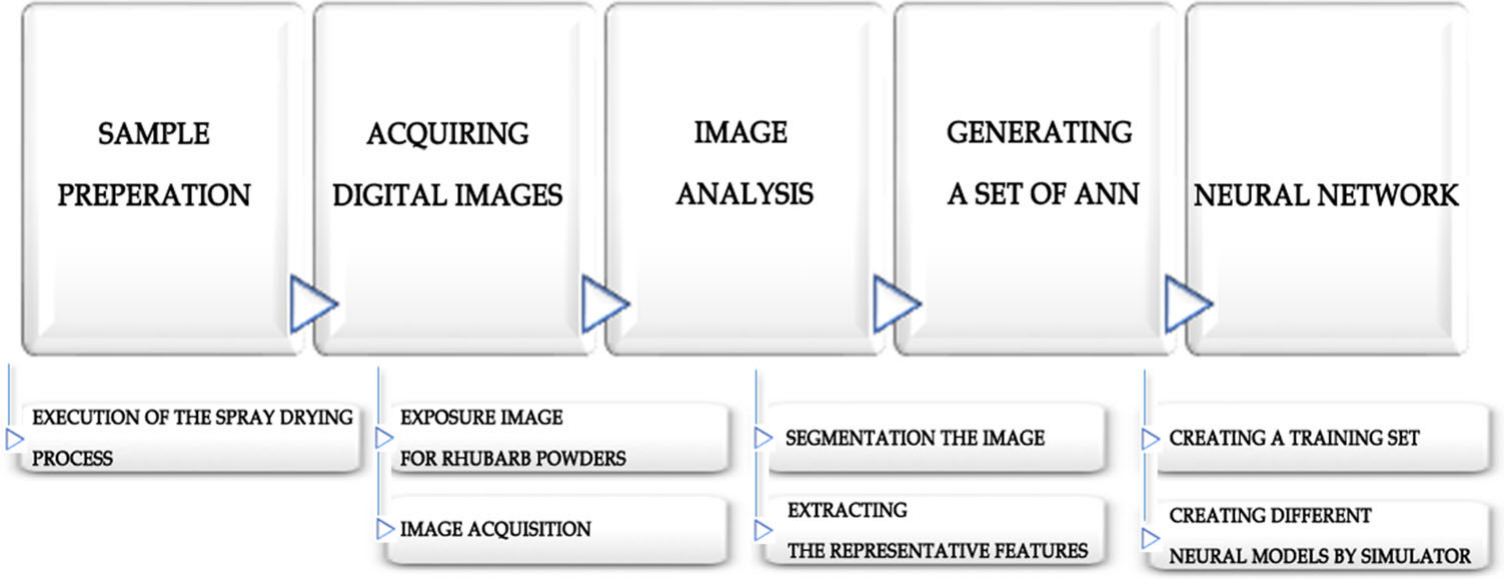
The research methodology scheme

In food industry, a lot of variables have influence on the appearance of food: i.e. size, mass, volume, color, uniformity, intensity, shine, shape and form, internal and external faults of product. In case of fruit and vegetable powders the crucial aspect is their texture and proper color, i.e. the power of dyeing. In order to conduct the research, the following tasks need to be carried out (Fig. 1):

- collecting material in the form of test samples of dried rhubarb juice, obtained through industrial spray drying processes intended for the processing liquid raw materials into powdery substances which largely recover the properties of the input material when dissolved in water,
- acquiring digital images of rhubarb powders,
- defining the characteristic features of rhubarb powders, depicted on digital images,
- developing a computer method of identifying and extracting the representative features (color space models: RGB, HSB, HSL, YCbCr),
- transforming the acquired empirical data into a form accepted by ANN simulators,
- creating a training set used to build neural models,
- generating a set of neural models and performing their initial assessment,
- identifying the optimal neural model with the greatest ability to recognize and classify rhubarb powders,
- validating and testing the generated neural model.

### Image analysis

**Fig. 2:**
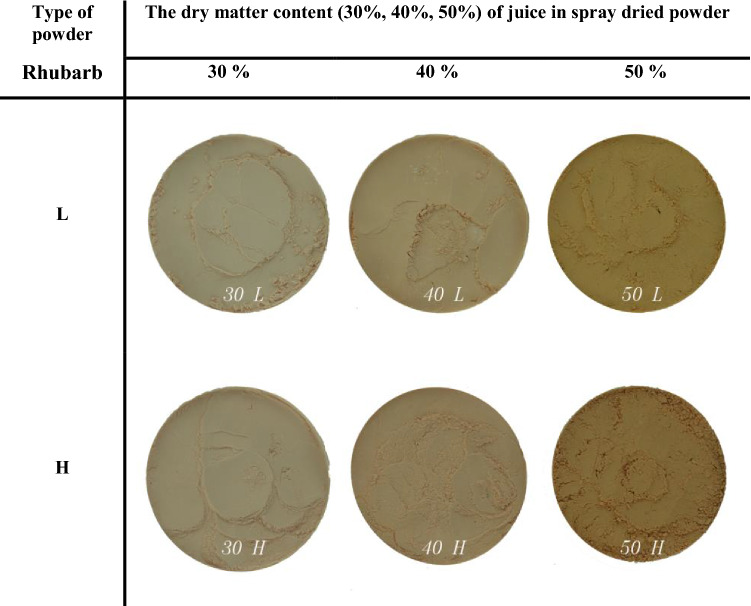
Typical color images for different dry matter juice content and the degree of saccharification carrier in spray dried rhubarb powder (“L”—low, “H”—high of the degree of saccharification carrier—dextrose equivalent of maltodextrine: “L”—DE 12, “H”—DE 26)

In order to identify the type of rhubarb powders: the dry juice content level and the degree of saccharification of used carrier (Fig. 2), a technique of image processing and analysis and a neural analysis of empirical data were used. The ‘PIDsystem’ software was used to support image processing. The ‘PIDsystem’ performed a series of steps in order to obtain the required information from digital photo in the models of color space such as: RGB (Prats-Montalbán et al. 2009; Kucheryavskiy 2013; Guzmán et al. 2015; Zareiforoush et al. 2016), YCbCr, HSB(V) (Khoje and Bodhe 2015) and HSL (Philipp and Rath 2002). The application isolated representative features of each image. By identifying and selecting those representative features of digital images (the so called discriminatory features), which were the most useful in the process of their distinguishing and classification, 46 adequate factors/coefficients were selected to build up a set of learning cases:

- R Max, R Mean, R Median, R Min, R Std—max, mean, median, min, standard deviation in the intensity of the Red component for the original image (5 features);
- G Max, G Mean, G Median, G Min, G Std—max, mean, median, min, standard deviation in the intensity of the Green component for the original image (5 features);
- B Max, B Mean, B Median, B Min, B Std—max, mean, median, min, standard deviation in the intensity of the Blue component for the original image (5 features);
- Y Max, Y Mean, Y Median, Y Min, Y Std—max, mean, median, min, standard deviation in the Y component for the image converted into YCbCr model (5 features);
- Cb Max, Cb Mean, Cb Median, Cb Min, Cb Std—max, mean, median, min, standard deviation in the Cb component for the image converted into YCbCr model (5 features);
- Cr Max, Cr Mean, Cr Median, Cr Min, Cr Std—max, mean, median, min, standard deviation in the Cb component for the image converted into YCbCr model (5 features);
- H Mean—mean in the Hue component for the image converted into HSV or HSL (1 feature);
- S Max, S Mean, S Median, S Min, S Std—max, mean, median, min, standard deviation in the Saturation component for the image converted into HSL or HSB model (5 features);
- B Max, B Mean, B Median, B Min, B Std—max, mean, median, min, standard deviation in the Brightness component for the image converted into HSV(B) model (5 features);
- L Max, L Mean, L Median, L Min, L Std—max, mean, median, min, standard deviation in the Luminance component for the image converted into HSL model (5 features).

In order to construct a learning set that includes 46 input variables, which constitute discriminatory parameters of color space models, a three-state output variable was defined. The output information consisted of answer to the question whether the material in the image determined the type of powder (No. Samples as TYPE variables and SUGAR) or the content of dry mass of juice in spray dried powder (TYPE variable) or whether it was possible to determine the level of saccharification (SUGAR).

### Artificial neural networks

In this case, the STATISTICA Neural Networks simulator was used. This tool offers modern programming techniques and has a lot of data analysis instruments, which support the process of generating ANN. Above all, it includes learning algorithms such as the fastest neighbor method, conjugate gradient method, Broyden–Fletcher–Goldfarb–Shanno (BFGS) method, Kohonen method, used for example in forming clusters and visualization of relations and k-means method for RBF networks (Kanungo et al. 2002). It also allows to chose between various activation functions, error functions and sizes of network structures. The training set generated with the ‘PIDsystem’ software contained 4218 training cases representing the aforementioned 46 variables. The set was divided in the simulator based on a ratio of 2:1:1, into the following subsets: training—2109 cases, validation—1054 cases and testing—1055 cases. The rejection threshold for the error quotient was set to 1.05, taking into consideration the relatively large number of input variables. The research were performed on the basis of the schedule guidelines, which indicated that it was possible to identify material found in the image, i.e. samples of fruit and vegetable powders. The Multi-Layer Perceptron network with the following structure: 46:46-11-10: 3 (Table 1) and with 46 neurons in the input layer (numerical data relating to the color), 11 neurons in the hidden layer and 10 neurons in the output layer (numerical data indicating the type of fruit powder) proved to be the optimum topology of the neuron network. The 46:46-11-10:3 MLP network was trained with algorithms including Back Propagation (BP) over 50 epochs, and then learned further with Conjugate Gradient (CG) algorithm in over 119 epochs.It should be noticed that that BP is a proven MLP network learning algorithm (Radhika and Rao 2015; Zareiforoush et al. 2016; Cheng et al. 2017). The algorithm allows for achieving satisfactory results thanks to features such as a high learning rate, a sequence of calculating signal of mistake size and possibility of generalization. MLP networks are characterized by being one-way networks where signal flows from input layer neurons to output layer neurons. Neurons included in MLP-type ANN networks aggregate input data by determining the sum of weighted outputs. This network uses a ‘teacher-assisted’ learning technique (Behbahani et al. 2014).

**Table 1:**
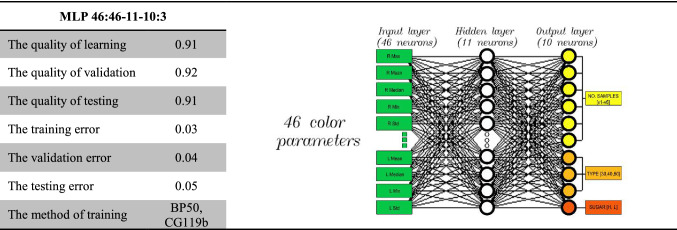
The results network training MLP 46:46-11-10:3

### Principal component analysis

In order to determine forecast, according to which, the best data regarding quality assessment of powders classified with rhubarb, the best option to is to present evaluation of classification with principal component analysis (PCA) analysis (Prats-Montalbán et al. 2009; Kucheryavskiy 2013). In this analysis, 15 of 46 selected variables based on a sensitivity analysis, which are most sensitive to the quality of learning ANN (CB_MEAN, HSV_HUE, CR_MEDIAN, G_STD, G_MEAN, Y_STD, CR_MIN, CB_MIN, CB_STD, CB_MAX, Y_MEAN, SATURATION_MEAN, CR_MEAN, R_STD, R_MEDIAN) laid out in the matrix of mean values for the learning set containing 4218 learning cases. Relationships between variables are performed with the help of correlation analysis. Values of own values are also assigned to 15 out of 46 representative features significant for the neural model and percentage of variance. It is determined, which variables have a significant correlation with each other to see (Ding et al. 2015; Ghosh and Chattopadhyay 2012; Nagaprabha and Bhattacharya 2016; Lyu et al. 2017) if these variables are capable of determining the quality level of fruit/vegetable juice powder and saccharification level of the used carrier.

## Results and discussion

### Results of classification

ANN are commonly used as classification instruments, in particular in several areas of food technology and agricultural engineering (Gatica et al. 2013). ANN can be used for analyzing many decision-making processes, forecasting, filtering and optimization of data (Murthy and Manohar 2014). ANN enable complete automatization and process control during all stages of process, from raw material through semi-finished good to ready made product. ANN are also used to support quality analysis of food products such as fruits and vegetables. One of the popular tools to design and create different neural models, apart from Matlab is simulator implemented as a package in STATISTICA.

In the last phase of research in STATISTICA, the following types of neural networks are tested: MLP and Radial Basis Function (RBF). 100 tests were done on account of selection of color discriminants from image, whose aim was to optimize training set, which in consequence meant ANN generation. Among 50 different ANN topologies that were generated, the network, which was characterized by the biggest classification abilities, was selected.

The measurement of correctness of network learning was checked on the basis of root mean square (RMS) error and classification accuracy. The RMS error created in the network generating process obtained low values (Table 1), at the same time it created adequate MLP model type 46:46-11-10:3. The low and similar RMS error value for each individual learning set indicated that the network had good classification abilities and the was no risk of the network learning again (Bishop 2006). It is calculated following the formula (1):

1 $$RMS = \sqrt {\frac{{\mathop \sum \nolimits_{i = 1}^{n} \left( {y_{i} - z_{i} } \right)^{2} }}{n}}$$

where *n*—number of cases, *y*_*i*_—real values, *z*_*i*_—values calculated with PNN network.

For optimal MLP 46:46-11-10:3 model created in the process of work, the RMS mistake amounted respectively:

- 0.03 for training set,
- 0.04 for validation set,
- 0.05 for testing set.

Network MLP 46: 46-11-10: 3 achieved high, satisfactory learning level for individual sets, i.e. at a level of approximately 0.92 (Table 1). Classification statistics regarding making mistakes, respectively in learning sets, validation sets and testing sets, which can be seen in Table 2, are confirmation of the results for a selected network. Information included in Table 2, show that good classification possibilities for a selected neural model are dependent on a minor mistake of RMS error both in learning and validation set. The above means that the network was not „overlearnt”.

**Table 2:** Classification statistics of learning sets: the training, the test, the validation

	Training file						Validation file						Test file					
	v1	v2	v3	v4	v5	v6	v1	v2	v3	v4	v5	v6	v1	v2	v3	v4	v5	v6
Total	348	365	382	333	341	340	155	174	190	179	173	183	171	175	166	172	172	199
Correct	319	326	364	305	284	331	145	160	185	168	137	178	154	162	157	149	147	191
Wrong	0	0	0	0	0	0	0	0	0	0	0	0	0	0	0	4	0	0
Unknow	29	39	18	28	57	9	10	14	5	11	36	5	17	13	9	19	25	8

	Training file			Validation file			Test file		
	rb_30	rb_40	rb_50	rb_30	rb_40	rb_50	rb_30	rb_40	rb_50
Total	681	706	722	334	347	373	343	347	365
Correct	681	706	722	333	347	373	342	347	365
Wrong	0	0	0	0	0	0	0	0	0
Unknow	0	0	0	1	0	0	1	0	0

	Training file		Validation file		Test file	
	L	H	L	H	L	H
Total	1095	1014	519	535	512	543
Correct	1049	968	501	511	492	509
Wrong	0	0	0	0	0	4
Unknow	46	46	18	24	20	30

In order to guarantee correctness of network learning process, it is important to pay attention to the quality of neural model in classification. This dependency results from such values, for which percentage of correct classification is determined (Table 2). The selected MLP network: 46: 46-11-10: 3, which was not „over-learnt” and was characterized by low RMS error, also reached quality level respectively:

- 0.91 for the quality of learning,
- 0.92 for the quality of validation,
- 0.91 for the quality of testing.

In order to determine efficiency of selected and adequate model, its accuracy classification is also tested. Accuracy classification depends on the number of correctly classified learning cases responsible for the efficiency of model forecasts and is calculated according to the following formula (Taylor 1997) (2):

2 $$Accuracy = \frac{t}{n}$$

where *t*—the number of sample cases correctly classified, *n*—number of cases.

In MLP model: 46:46-11-10:3 accuracy classification for input variables amounts respectively:

- 0.92 of No. Sample in spray dried rhubarb powder,
- 0.99 of dry matter juice content in spray dried rhubarb powder,
- 0.96 of the degree of saccharification carrier in spray dried rhubarb powder.

After conducting the analysis on: No. Sample in spray dried rhubarb powder, dry mass juice content in spray dried rhubarb and the degree of carrier saccharification in spray dried rhubarb powder, it turned out that the highest degree of classification efficiency was obtained in dry mass content in spray dried rhubarb powder. Nevertheless, as a selected neural model, it was characterized by high accuracy of classification for input variables, which confirmed its efficiency in recognizing spray dried rhubarb juice. As part of comparison process, the authors demonstrated in other research efficiency of quality identification of chokeberry powders on account of their highest dyeing power, the highest bioactivity as well as technologically satisfying looseness of powder.

After conducting a series of research, the authors noticed that MLP was the most effective typology of networks recognizing quality discriminants of powders including rhubarb powder, chokeberry powder (Przybył et al. 2019) and strawberry powder (Przybył et al. 2018). Research to date, conducted by the authors, on fruit powders, were aimed at obtaining above all on the basis of quality quality discriminants of homogeneity and repetitiveness of obtaining fruit and vegetable powders.

### Results of analysis of sensitivity to input variables

The last phase defined the level of significance of individual parameters used for the construction of the neural classifier with the use of analysis of sensitivity to input variables. Sensitivity analysis allowed to determine the usefulness of various input variables. Variables that had a high priority might be omitted due to the fact that they did not affect the quality of the teaching model. However, variables characterized by low-ranking and high quotient of error must not be overlooked. Loss of low-rank high error quotient variables significantly affected the sensitivity of the generated neural model (Table 3). Table 3 shows 15 of 46 variables relating to color space models where the lowest rank occurred. Analysis showed that the 5 lowest variables occurred in respect of the following variables: CB_MEAN—specifying the average for component difference of the illumination and the color blue in YCbCr model, HSV_HUE—defining the average component of hue, which had a value ranging 0˚ ÷ 360˚ in HSV model (Salehi and Kashaninejad 2015), CR_MEDIAN—defining the median of the component difference of the illumination and the color red, G_STD and G_MEAN—determining the standard deviation and the average components of green color RGB model (Table 3). The highest rank of variable occurred in respect of the following variable: Y_MAX—specifying the maximum for component of the light intensity (luminance) in YCbCr model (Table 3). An artificial neural network was sensitive to the absence of the input variable whose quotient was greater than 1, which results in deterioration of the quality of learning the neural network. The high level of significance of 46 variables played a dominant role in the identification of types of spray dried rhubarb juice.

**Table 3:** The results of sensitivity analysis

Rank	Variable	Quotient
1	CB_MEAN	7.15
2	HSV_HUE	5.30
3	CR_MEDIAN	3.97
4	G_STD	3.72
5	G_MEAN	3.38
6	Y_STD	3.27
7	CR_MIN	2.91
8	CB_MIN	2.76
9	CB_STD	2.71
10	CB_MAX	2.58
11	Y_MEAN	2.46
12	SATURATION_MEAN	2.44
13	CR_MEAN	2.44
14	R_STD	2.30
15	R_MEDIAN	2.26
⋮	⋮	⋮
45	Y_MIN	1.02
46	Y_MAX	1.00

### Results of principal component analysis

The analysis of sensitivity to input variables showed certain correlations (Table 4). Correlation between R, G, and B values from encoded information of graphical objects of the RGB model was found using computer image analysis. A strong correlation was found between R Median and G Mean value from the RGB model; whereas for R Median and G Std value, the correlation coefficient was lower. A strong correlation was found in the YCbCr model between Cb Mean and Y Mean values; whereas the correlation coefficient in respect of Y Mean and Cr Mean values was lower.

**Table 4:** The correlation matrix of analysis of sensitivity

	HSV Hue	R Median	R Std	G Mean	G Std	Y Mean	Y Std	Cb Max	Cb Std	Cb Mean	Cb Min	Cr Mean	Cr Median	Cr Min	Saturation Mean
HSV Hue	1.00														
R Median	0.67	1.00													
R Std	− 0.74	− 0.68	1.00												
G Mean	0.79	0.96	− 0.81	1.00											
G Std	− 0.70	− **0.60**	0.97	− 0.72	1.00										
Y Mean	0.77	0.97	− 0.80	1.00	− 0.71	1.00									
Y Std	− 0.72	− 0.61	0.99	− 0.74	1.00	− 0.73	1.00								
Cb Max	− 0.09	− 0.31	0.21	− 0.21	0.31	− 0.21	0.27	1.00							
Cb Std	− 0.32	− 0.58	0.47	− 0.53	0.58	− 0.54	0.54	0.72	1.00						
Cb Mean	0.51	0.72	− 0.57	0.80	− 0.49	0.82	− 0.51	0.06	− 0.36	1.00					
Cb Min	0.50	0.76	− 0.64	0.83	− 0.58	0.84	− 0.59	− 0.05	− 0.48	0.97	1.00				
Cr Mean	− 0.82	− 0.85	0.78	− 0.95	0.70	− **0.95**	0.72	0.05	0.42	− 0.89	− 0.89	1.00			
Cr Median	− 0.81	− 0.87	0.77	− 0.96	0.71	− 0.95	0.72	0.13	0.53	− 0.88	− 0.88	0.99	1.00		
Cr Min	− 0.28	− 0.20	0.29	− 0.31	0.12	− 0.30	0.17	− 0.75	− 0.44	− 0.39	− 0.31	0.40	0.31	1.00	
Saturation Mean	− 0.67	− 0.91	0.78	− 0.96	0.69	− **0.97**	0.71	0.14	0.52	− 0.92	− 0.94	0.96	0.96	0.35	1.00

Ultimately, the strongest correlation in the YCbCr model was found between Cb Mean values and Cb Min values, and amounted to r^2^ = 0.97; another strong correlation was found between Cr Median and Cr median values; amounting to r^2^ = 0.99, whereas the correlations between R Std and G Std values in the RGB model amounted to r^2^ = 0.97. It was found that among the 46 input variables important to the quality of the neural model, the strongest correlation was present between the component for the luminance of mean value (Y Mean) in the YCbCr model and the component for the color green of mean (G Mean) in the RGB model. In the case of model HSB or HSL coefficients, a strong correlation was found between information about saturation values (Saturation Mean) and Cr values (Cr Median or Cr Mean); while in the case of saturation value (Saturation Mean) and luminance value (Y Mean), the correlation coefficient was the lowest.

**Fig. 3:**
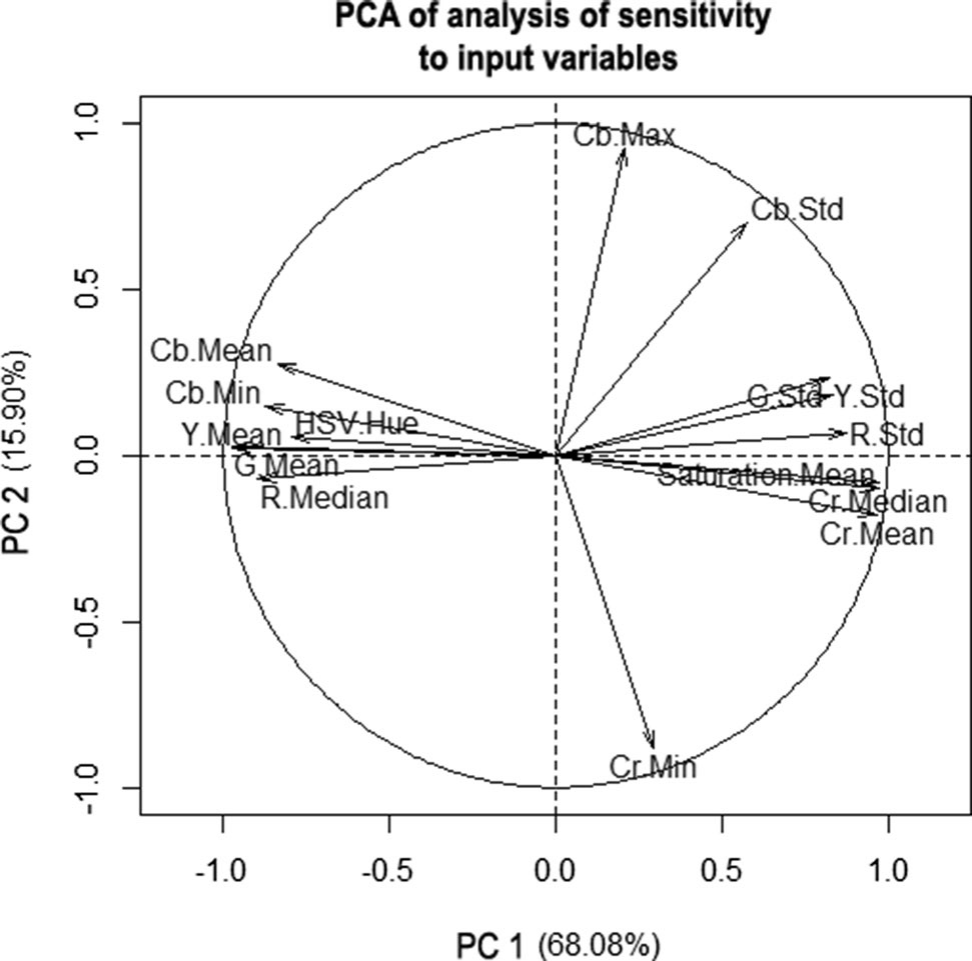
Location vectors 15 with 46 of input variables on basis of terms of the first two principal components

The PCA was performed (Fig. 3) and eigenvalues were set (Table 5). These values could be used to determine the percentage variation of the samples; a score of 68.08% on the first principal component (PC1) and a score of 15.09% for the second principal component (PC2) were obtained, and a total score of 83.98%. The first two principal components “transferred” over 83% of the variability of the original data. It was possible to analyze the original dataset in only two dimensions with good approximation (Table 5). The input variables perpendicular to each other were not correlated (for instance, HSV Hue value between R Std value). The Cr Median and Cr Mean values, which can be seen on the chart, were close to each other, which means that they were strongly positively correlated. The Cr Mean and Cb Mean variables were on opposite sides, which means they are strongly negatively correlated.

**Table 5:** Eigenvalues

	PC1	PC2	PC3	PC4	PC5	PC6	PC7	PC8	PC9	PC10
Variance	10.21	2.39	1.21	0.56	0.32	0.19	0.08	0.02	0.01	0.01
% of var.	68.08	15.90	8.05	3.70	2.13	1.27	0.53	0.15	0.08	0.06
Cumulative % of var.	68.08	83.98	92.03	95.73	97.87	99.14	99.66	99.81	99.90	99.95

## Conclusion

The development of civilization led to emergence of various drying methods with artificial drying factor. Many factors influenced the above development, among other things, product diversity so designing the most optimal drying method, for which it is possible to preserve as many nutritional values as possible. Endeavors were made to preserve season products to make the available all year round. That is why, searching for methods assessing quality and methods of preserving products seem to justified.

The use of techniques of processing operations and digital image analysis, and method of artificial neural networks allowed the adequate identification of the type and concentration of the research material, i.e. spray dried rhubarb juice with varying level of dry juice content in the powders and varying degrees of saccharification of the used carrier in spray drying process.

The qualitative analysis demonstrated that the adequate neural model which obtained the highest classification capability was found in a network of MLP, which had the following structure: 46: 46-11-10: 3. The generated neural model of type MLP-46:46-11-10:3 reached an RMS error value of 0.04.

Analysis of sensitivity to input variables for MLP 46:46-11-10:3 demonstrated that the most important variable was the component for the difference in light intensity and the color blue (CB_MEAN). The most important of the 15 representative features were the descriptors of the color space of model YCbCr with the following rank: 1, 3, 7-11, 13,14.
